# Role of N6-methyladenosine RNA modification in gastric cancer

**DOI:** 10.1038/s41420-023-01485-z

**Published:** 2023-07-13

**Authors:** Si-Qi Ding, Xue-Ping Zhang, Jun-Peng Pei, Xiao Bai, Jin-Jie Ma, Chun-Dong Zhang, Dong-Qiu Dai

**Affiliations:** 1 Department of Gastrointestinal Surgery, The Fourth Affiliated Hospital of China Medical University, 110032 Shenyang, China; 2 Cancer Center, The Fourth Affiliated Hospital of China Medical University, 110032 Shenyang, China

**Keywords:** Cancer epigenetics, Targeted therapies, Cancer metabolism, Gastric cancer

## Abstract

N6-methyladenosine (m6A) RNA methylation is the most prevalent internal modification of mammalian messenger RNA. The m6A modification affects multiple aspects of RNA metabolism, including processing, splicing, export, stability, and translation through the reversible regulation of methyltransferases (Writers), demethylases (Erasers), and recognition binding proteins (Readers). Accumulating evidence indicates that altered m6A levels are associated with a variety of human cancers. Recently, dysregulation of m6A methylation was shown to be involved in the occurrence and development of gastric cancer (GC) through various pathways. Thus, elucidating the relationship between m6A and the pathogenesis of GC has important clinical implications for the diagnosis, treatment, and prognosis of GC patients. In this review, we evaluate the potential role and clinical significance of m6A-related proteins which function in GC in an m6A-dependent manner. We discuss current issues regarding m6A-targeted inhibition of GC, explore new methods for GC diagnosis and prognosis, consider new targets for GC treatment, and provide a reasonable outlook for the future of GC research.

## Facts

- N6-methyladenosine RNA methylation affects RNA metabolism in gastric cancer.
- Dysregulation of m6A participates in the occurrence and development of gastric cancer.
- The role and significance of m6A regulators that function in gastric cancer in an m6A-dependent manner.
- The clinical value and current problems of m6A in gastric cancer.

## Open questions

- How do m6A regulators influence the progression of gastric cancer in an m6A-dependent manner?
- Could develop novel methods for gastric cancer diagnosis and prognosis prediction with m6A as the target?
- Could existing m6A targeted inhibitors be applied to gastric cancer, and if not, how could they be improved?

## Introduction

Gastric cancer (GC) is the fourth main cause of cancer-related death and fifth most diagnosed cancer worldwide [1]. Although GC deaths have decreased globally, the prevalence of GC in East Asia (including China) is still high [2]. GC is ranked second in cancer deaths in both Chinese men and women, with a very low 5-year survival rate [3]. For patients with early GC (EGC), the 5-year cancer-specific survival (CSS) rate with timely treatment can exceed 95% [2, 4]. However, due to a lack of early symptoms and a low rate of gastroscopy, most patients have progressed to an advanced disease stage when initially diagnosed [3, 5]. The patients with advanced GC whose 5-year survival rate is less than 10% [6], with 79% of GC patients facing recurrence and metastasis within two years, even after surgical resection [7]. The effective rate for chemotherapy and combined therapy for advanced GC is approximately 40% [8, 9]. Since recurrence and metastasis significantly affect GC patient prognosis, there is an urgent to explore the mechanistic basis for recurrence and metastasis, develop new modalities for early diagnosis, increase survival rates, and improve the prognosis for GC patients.

Epigenetics describes heritable changes in gene expression that do not involve changes in the nucleotide sequences of genes. An important form of epigenetic modification is n6-methyladenosine (m6A) RNA modification, which was first discovered in eukaryotic cells in 1974 by Desrosiers et al. [10]. In 2011, the investigation into the fat mass and obesity-related protein (FTO), the first m6A demethylase was identified and found to reverse m6A RNA methylation, indicating that m6A RNA methylation was dynamic and reversible [11]. With the emergence of powerful analytical methods, m6A-seq [12] and MeRIP-seq [13], m6A RNA modification could be fully explored. Other m6A detection methods include two-dimensional thin layer chromatography [14], dot-blot [11], high-performance liquid chromatography coupled to triple-quadrupole mass spectrometry (LC–MS/MS) [15], photo-crosslinking-assisted m6A-sequencing (PA-m6A-Seq) [16], site-specific cleavage and radioactive-labeling followed by ligation-assisted extraction and thin-layer chromatography (SCARLET) [17], as well as m6A individual nucleotide resolution crosslinking immunoprecipitation (miCLIP) [18].

Continued advances in detection and sequencing technology, have led to the recognition that m6A modification is the most prevalent and important form of internal methylation of mammalian mRNA [19–21]. Previous studies have shown that dysregulation of m6A is associated with various cancer biological processes (RNA processing, splicing, stability, and translation) [21] and may be involved in the initiation and progression of multiple cancers [22].

Recently, the study of GC epigenetics has intensified, with m6A RNA methylation demonstrated to be involved in epigenetic regulation of GC, especially in carcinogenesis and tumor progression. Emerging evidence suggests that m6A dysregulation may participate in a variety of GC biological processes through many pathways [23–25]. Therefore, elucidating the relationship between m6A and the pathogenesis of GC is important for GC early diagnosis, treatment, and patient prognosis. We pay close attention to the function of m6A in GC and the clinical significance of this epigenetic modification. We also provide recommendations for the focus of future investigations.

## Foundation of m6A RNA methylation

**Fig. 1:**
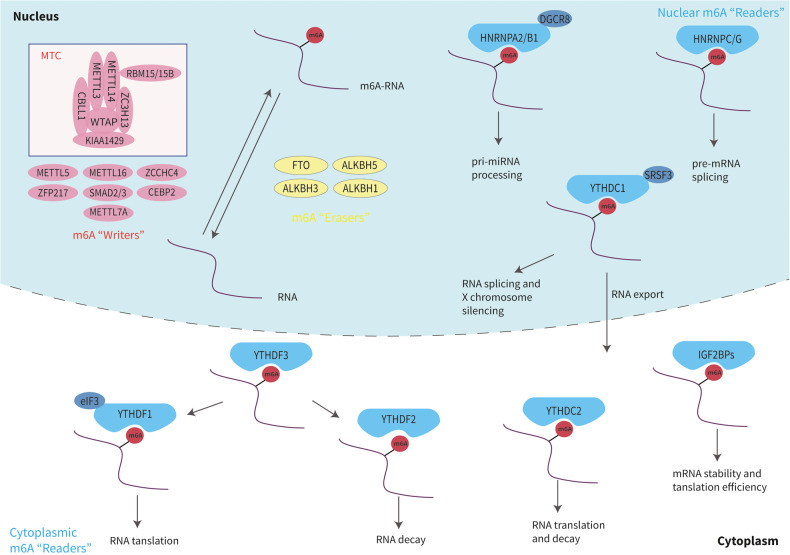
Working mechanism of m6A methylation. “Writers” catalyze RNA to m6A-RNA. “Erasers” reverse m6A-RNA to RNA. “Readers” in the nucleus mediate RNA processing, splicing and X chromosome silencing. “Readers” in the cytoplasm mediate RNA stability, translation, and decay.

M6A modification requires three types of regulatory proteins: “Writers” (methyltransferases), “Erasers” (demethylases), and “Readers” (recognition binding proteins) [23]. Among these, Writers are responsible for catalysis of the m6A modification. Erasers are responsible for reversal of m6A modification, while Readers recognize the m6A modification [21] (Fig. 1). M6A modification is strictly restricted to the common sequence “RRACH” ([R = A or G] m6AC [H = U or C or A]), and enriched in regions close to 3’-UTRs of mRNAs, or the last exon of noncoding RNAs (ncRNAs) [12, 13, 25, 26].

## Writers

Writers are a class of methyltransferases with the ability to catalyze m6A formation. The methyltransferase complex (MTC) is comprised of methyltransferase-like 3-methyltransferase-like 14 (METTL3-METTL14) heterodimer, Wilms tumor 1-associated protein (WTAP), Vir-like m6A methyltransferase associated (VIRMA/KIAA1429), RNA binding motif protein 15 (RBM15) and its collateral homolog, RNA binding motif protein 15B (RBM15B), Cbl proto oncogene like 1 (CBLL1 or HAKAI), and zinc finger CCCH-type containing 13 (ZC3H13) [27–29]. Among them, METTL3 that uses S-adenosyl methionine (SAM) as the methyl donor, is the catalytic core for m6A MTC [25, 27]. METTL14 provides structural support for METTL3 and forms a stable heterodimer core complex with METTL3. This METTL3-14 dimer can mediate the deposition of m6A on mammalian nuclear RNAs [20]. WTAP does not possess methylation activity but does interact with the METTL3-14 complex to stabilize the core complex [30, 31]. RBM15/RBM15B mediates m6A formation in both XIST and cellular mRNAs by binding to MTC and recruiting it to specific RNA sites [32]. KIAA1429 (VIRMA) selectively guides methylations by recruiting MTC to 3’UTRs and to near stop codons [28]. Emerging evidence suggests that ZC3H13 [29, 33], a novel cofactor for the m6A MTC, plays a key role in RNA m6A methylation, where it mediates the nuclear localization of the ZC3H13-WTAP complex. YUE et al. [28] proposed that HAKAI (CBLL1) and possibly WTAP/ZC3H13 use KIAA1429 (VIRMA) as a scaffold to form a suitable pocket for METTL3/METTL14, which uses WTAP to guide m6A modifications at 3’UTRs, near stop codons.

Another m6A Writer, methyltransferase-like 16 (METTL16) [34] was found to be a methyltransferase of the U6 spliceosomal small nuclear RNA (snRNA) that regulates SAM homeostasis with a catalytic substrate of a “UACAGAGAA” sequence. Methyltransferase-like 7A (METTL7A), a novel methyltransferase, contains an *S*-adenosylmethionine domain [35] that promotes m6A methylation of lncRNA in adipocytes. This methylation allows for enrichment within adipocyte exosomes [36]. Methyltransferase-like 5 (METTL5) is the enzyme responsible for m6A modification of 18S rRNA, while ZCCHC4 is responsible for 28S rRNA m6A modification. They deposit m6A into their respectively responsible rRNAs [37]. Besides, there are some transcriptional factors involved in m6A deposition on mRNAs, including zinc finger protein 217 (ZFP217) [38], SMAD2/3 [39], and the CAAT box-binding protein (CEBPZ) [40].

## Erasers

Demethylases with the ability to remove m6A modifications are known as Erasers of m6A methylation. Known demethylases include fat mass and obesity associated (FTO) protein and AlkB homologue 1/3/5 (ALKBH1/3/5). Jia et al. discovered the first m6A demethylase, FTO, in 2011 [11]. ALKBH5 was discovered in 2013 as a second demethylase by Zheng et al. [41].

Both FTO and ALKBH5 belong to the AlkB family of Fe (II)/a-ketoglutarate (a-KG)-dependent dioxygenases that reverse m6A in nuclear speckles [23, 27, 29, 30]. ALKBH1 was previously considered a DNA and tRNA demethylase [42, 43]. More recently, ALKBH1 was found to promote the cell invasion and migration of lung cancer by regulating RNA m6A levels [44]. ALKBH3 was also found to act as an m6A demethylase of mammalian transfer RNA (tRNA). The protein translation efficiency of tRNA is improved with ALKBH3 modification [45].

## Readers

Proteins that specifically recognize and bind to m6A modification sites are known as Readers, including YT521-B homology (YTH) domain family proteins 1/2/3 (YTHDF1/2/3), YTH domain-containing proteins 1/2 (YTHDC1/2), insulin-like growth factor 2 mRNA-binding proteins (IGF2BP) 1/2/3 (IGF2BP1/2/3), eukaryotic translation initiation factor 3 (eIF3), and members of the heterogeneous nuclear ribonucleoprotein (HNRNP) family (HNRNPA2/B1, HNRNPC, and HNRNPG) [23, 25, 46]. The readers (YTHDC1 and members of the HNRNP family) are localized in the nucleus and the other readers are localized in the cytoplasm [29]. Readers with differing cellular positions generate different functional signals by decoding m6A and regulating downstream biological function. Previous studies showed that YTHDC1 has multiple functions, including promotion of XIST-mediated X chromosome silencing [32], nuclear export of m6A methylated transcripts [47], and exon inclusion in targeted mRNAs via interaction with two splicing factors SRSF3 (SRp20) and SRSF10 (SRp38) [48]. Recent studies found YTHDC1 and its target m6A RNAs, within mouse embryonic stem cells, avoid cellular reprogramming at the two-cell (2C)-like stage and the reactivation of silenced retrotransposons [49]. With the aid of a unique protein called DGCR8, HNRNPA2/B1 exerts a positive effect on the processing of primary microRNA (pri-miRNA) by binding to m6A of pri-miRNA transcripts [50]. HNRNPC and HNRNPG were found to be involved in the formation of the “m6A switch” phenomenon by mediating pre-mRNA splicing to alter the secondary structure of RNAs [51, 52].

Readers in the cytoplasm also play an important biological role. For example, through combining with m6A, YTHDC2 can reduce the abundance of mRNA and improve the translation of its targets. YTHDC2 may also play a role in early spermatogenesis and is essential for proper spermatocyte development [53]. YTHDF1, YTHDF2 and YTHDF3 contain YTH domains that are highly homologous [54], playing completely different cellular roles. YTHDF1 improves the translation efficiency of target RNAs by binding to m6A sites near the stop codon of mRNAs as well as interacting with translation initiation complexes that contain eIF3 [55]. YTHDF2, the first identified m6A Reader [25], promotes the decay of its targets by combining with the CCR4-NOT complex and recognizing specific m6A sites [56]. YTHDF3 has two functions: cooperating with YTHDF1 to promote the translation of m6A-containing RNAs, and interacting with YTHDF2 to accelerate target mRNAs decay and degradation [57, 58]. IGF2BP1/2/3-mediated m6A improves the stability and translation efficiency of target mRNAs by recognition of the GG (m6A) C sequence [59]. Known m6A modification regulators are identified in Table 1.

**Table 1:** The function of m6A regulators in RNA metabolism^a^.

Type	Regulator	Function	Reference
Writers	METTL3	catalyzes m6A formation	[25, 27]
	METTL14	combines METTL3 and provides structural support for METTL3	[20]
	WTAP	interacts with METTL3-METTL14 and stabilizes the core complex (MTC)	[30, 31]
	KIAA1429 (VIRMA)	guides MTC to specific RNA regions and guides region-selective methylation	[28]
	RBM15/15B	binds the m6A complex and recruits it to specific RNA site	[32]
	HAKAI (CBLL1)	binds to WTAP-ZC3H13 and using KIAA1429 as a scaffold forms a pocket suitable for MTC	[28]
	METTL16	catalyzes m6A formation	[34]
	METTL7A	catalyzes m6A formation	[35, 36]
	ZC3H13	binds to WTAP and assists with MTC nuclear localization	[29, 33]
	METTL5	deposits m6A into 18S rRNA	[37]
	ZCCHC4	deposits m6A into 28S rRNA	[37]
	ZFP217	participates in m6A deposition of mRNA	[38]
	SMAD2/3	participates in m6A deposition of mRNA	[39]
	CEBPZ	participates in m6A deposition of mRNA	[40]
Erasers	FTO	removes m6A modification	[23, 27, 29, 30]
	ALKBH5	removes m6A modification	[23, 27, 29, 30]
	ALKBH1	removes m6A modification	[43, 44]
	ALKBH3	removes m6A modification	[45]
Readers	YTHDC1	promotes RNA splicing and nuclear export	[32, 47, 48]
	YTHDC2	improves the translation efficiency of target mRNA and reduces the abundance of target mRNA	[53]
	YTHDF1	promotes mRNA translation	[55]
	YTHDF2	promotes mRNA degradation	[56]
	YTHDF3	promotes mRNA translation and degradation by interacting with YTHDF1 and YTHDF2	[57, 58]
	IGF2BP1/2/3	improves mRNA stability and translation efficiency	[59]
	HNRNPA2/B1	promotes pri-miRNA processing	[50]
	HNRNPC/G	mediates mRNA splicing	[51, 52]
	EIF3	enhances mRNA translation	[55]

^a^The function of m6A regulators in RNA metabolism is summarized.

## M6A effectors in GC

### METTL3 in GC

METTL3 is the most studied and important m6A methyltransferase, “Writer”, which plays a key role in a variety of cancers (including GC) [27].

#### METTL3 in GC growth, proliferation, migration, and invasion

The growth, proliferation, migration, and invasion of tumor cells are the basic malignant behaviors of cancer cells. Increasing evidence suggests that METTL3 affects the malignant behaviors of tumor cells and GC prognosis.

Li et al. [60] found METTL3-knockdown to attenuate the regulation of suppressor of cytokine signaling 2 (SOCS2) RNA stability via m6A modification, reducing the decay rate of SOCS2 RNA, which significantly inhibited the proliferation of GC cells. Moreover, METTL3 can m6A modify YES-associated protein 1 (YAP1) mRNA of the Hippo pathway, facilitating expression, and thus, GC proliferation and migration [61]. Recently, a new study [62] indicated that METTL3 boosts GC proliferation, colony formation, migration, and invasion in an m6A-dependent manner through the pre-B-cell leukemia homeobox 1 (PBX1)/GTP cyclohydrolase 1 (GCH1)/tetrahydrobiopterin (BH4) axis.

Many studies have shown that m6A and noncoding RNAs (ncRNAs) can interact and affect cancer progression [63]. NcRNAs are RNAs that do not have protein-encoding functions and include miRNAs, lncRNAs, circRNAs and pri-miRNAs [64]. METTL3 effects on GC progression may require additional m6A regulatory proteins. For example, with the aid of METTL3, the lncRNA LINC02253 increases KRT18 mRNA stability. KRT18 then positively regulates GC growth and migration through the MAPK/ERK signaling pathway [65]. The microRNA miR-4429 targets and downregulates the expression of METTL3. The result is a reduction in the stabilizing effect of IGF2BP1 on pre-protein translocation factor (SEC62) mRNA, inhibiting the expression of SEC62, the proliferation of GC cells, and increasing apoptosis [66]. The lncRNA THAP7-AS1 is transcriptionally activated by transcription factor SP1, which is enhanced by METTL3 mediated m6A modification that depends on the IGF2BP1 pathway, promoting GC growth, migration, and invasion through the PI3K/AKT pathway [67]. Huo et al. [68] demonstrated that METTL3 promotes the malignant biological behaviors of GC cell via the sphingosine kinase 2 (SPHK2)/Kruppel-like factor 2 (KLF2) axis in an m6A-YTHDF1-dependent manner. High levels of GC expression of SPHK2 predict a poor prognosis for GC patients. These results suggest that high levels of METTL3 expression may affect GC malignant progression.

#### METTL3 in GC chemo-resistance

Combined chemotherapy is currently one of the main treatment methods for advanced GC patients [8]. The drug resistance of some GC cells to chemotherapeutic drugs is the main drawback to GC chemotherapy and is the reason for the low efficiency of treatment for GC patients. METTL3 may be a potential indicator for treating chemo-resistant GC cells.

High levels of the lncRNA ARHGAP5-AS1 induce m6A modification of ARHGAP5 mRNA by recruiting METTL3, which upregulates ARHGAP5 and induces chemo-resistance that is related to poor prognosis in GC patients [69]. Moreover, in CD113 + GC stem cells, METTL3 increases PARP1 stability by recruiting YTHDF1 to specific m6A modification sites of poly (ADP-ribose) polymerase 1 (PARP1) mRNA. PARP1 then mediates DNA damage repair by base excision repair (BER), promoting oxaliplatin resistance in CD133 + GC stem cells [70].

#### METTL3 in GC angiogenesis

Angiogenesis is critical for rapid proliferation and distant metastasis of tumor cells. Thus, angiogenesis is essential for tumor initiation and progression. Inhibition of METTL3 expression decreases tumor cell angiogenesis, which inhibits the distant metastasis of GC and delays cancer progression. Wang et al. [71] found that high levels of METTL3 can strengthen hepatoma-derived growth factor (HDGF) mRNA stability by cooperating with m6A-dependent IGF2BP3. Partially upregulated HDGF induces GC angiogenesis. Moreover, METTL3 to reduce ADAMTS9 expression in an YTHDF2-mediated m6A manner, suppressing expression and accelerating GC angiogenesis [72].

#### METTL3 in GC metastasis and epithelial-mesenchymal transition (EMT)

Tumor metastasis is associated with unfavorable patient outcomes and a high degree of mortality [73]. EMT is the key to cancer metastasis. The downregulation of epithelial cadherin (E-cadherin) expression is usually indicative of EMT progression [74]. Accumulative evidence indicates that m6A participates in GC metastasis and EMT.

In GC, overexpressed METTL3 enhances GC cell glycolysis in an m6A-dependent manner through the HDGF/GLUT4/ENO2 pathway, thereby promoting GC proliferation and liver metastasis [71]. METTL3 has been shown to activate the AKT/mTOR pathway by targeting the inhibition of PTEN/TMEM127 expression through m6A modification at the pri-mir-17-92 A879 site, promoting growth and metastasis of GC [75]. Moreover, since METTL3 activates the mTOR pathway in GC cells, cells with high levels of METTL3 expression had a high degree of sensitivity to the mTOR inhibitor, everolimus. Further, silencing hepatitis B X interacting protein (HBXIP) reduced MYC expression by METTL3-mediated c-myc proto-oncogene mRNA m6A modification, which inhibited the malignant progression of GC, as well as increased cell apoptosis [76].

METTL3-dependent m6A modification is essential for EMT and GC metastasis. METTL3 downregulates the expression of E-cadherin through zinc finger MYM-type containing 1(ZMYM1)-CtBP-LSD1-CoREST complex, which activates EMT and metastasis of GC [77]. Likewise, homeobox A10 (HOXA10), partly through METTL3-mediated m6A modification, activates TGFβ/Smad pathway and downregulates the expression of E-cadherin, thereby inducing GC EMT progression and metastasis [78].

In conclusion, METTL3 regulates GC progression through various pathways. We found that METTL3 is an oncogenic factor of GC, and usually highly expressed in GC, with increased expression indicative of poor patient prognosis and a higher degree of malignancy. Therefore, a deeply study of the role of METTL3 in GC will provide a foundation upon which to build methods to detect and to target treatments for GC that will be of high clinical diagnostic value.

### METTL14 in GC

METTL14 may also influence GC progression by regulating the expression of ncRNAs. METTL14 enhances LINC01320 RNA stability and promotes expression through m6A-dependent modification, with subsequently overexpressed LINC01320 promoting GC cell proliferation, migration, and invasion through regulation of the miR-495-5p/RAB19 axis [79]. Obviously, METTL14 promotes GC. However, further investigation is necessary to fully address this issue.

Another study [80] found METTL14 to be poorly expressed in GC, which was associated with unfavorable patient outcomes. Ectopic expression of METTL14 significantly inhibited GC progression. Mechanistically, METTL14 induces m6A-dependent modification of circORC5, which suppresses expression and subsequently inhibits GC growth, proliferation, and invasion through regulation of the miR-30c-2-3p/AKT1S1 axis. As such, METTL14 may have a completely opposite role with different ncRNAs, suggesting that different m6A modification sites may influence the outcomes of METTL14-dependent m6A modification.

In summary, the role of METTL14 in GC is controversial, and specific mechanisms of action require further investigation.

### WTAP, KIAA1429 (VIRMA), and METTL16 in GC

Evaluation of a role for the m6A methyltransferase, WTAP, in GC tumorigenesis is in an early discovery stage. Current published studies have shown WTAP to act as an oncogenic factor for GC. Li et al. [81] found WTAP overexpression to be associated with RNA methylation and to be associated with poor GC prognosis. A more recent study [82] demonstrated that WTAP and m6A were highly expressed in GC. WTAP promoted GC cell proliferation and tumor growth. Further, WTAP promotes aerobic glycolysis (the Warburg effect) (including glucose uptake, lactate production, and extracellular acidification rate) of GC cells by targeting hexokinase-2 (HK2) in an m6A-dependent manner.

Currently, a single article has clearly reported that KIAA1429 (VIRMA), an m6A methyltransferase, promotes tumorigenesis in GC via m6A-dependent methylation. KIAA1429 has been shown to be highly expressed in GC samples. KIAA1429 stabilized LINC00958 and accelerated GC aerobic glycolysis by targeting the glucose transporter-1 (GLUT1) in an m6A-dependent manner, ultimately promoting GC progression [83].

As a new methyltransferase “Writer”, METTL16 had also been found to promote the progression of GC. METTL16 was shown to be upregulated in GC and to predict an unfavorable outcome. METTL16-mediated m6A methylation was found to regulate the GC cell cycle by enhancing cyclin D1 (cyclinD1) mRNA stability and increasing expression, thus facilitating GC cell proliferation. Further, downregulation of METTL16 significantly inhibited the proliferation of GC by restricting the cell cycle to the G1/S phase. METTL16 has also been found to facilitate tumor growth [84].

### FTO in GC

FTO is a critically important demethylase. It is reported to regulate GC RNA metabolism by reducing m6A methylation levels, thus, acting as an oncogenic factor and serving as a critical danger element for the prognostic of GC patients. This may be related to the following regulatory mechanisms.

Histone deacetylase 3 (HDAC3) promotes the progression of GC by regulating the transcription factor FOXA2-mediated FTO/m6A/MYC signaling pathway [85]. In addition, FTO is highly expressed in GC in which its expression reduces m6A modification degree of HOXB13. Consequently, high levels of HOXB13 promote GC cell proliferation, migration, and invasion through the PI3K/AKT/mTOR pathway [86]. A recent study [87] found that FTO directly binds to an m6A modification site on caveolin-1 mRNA to facilitate its degradation, thus, promoting the malignant biological behaviors of GC cells by regulating mitochondrial fission/fusion and metabolism (inducing mitochondrial respiration to increase ATP supplementation).

Furthermore, FTO has also related to chemo-resistance and apoptosis of GC. Proton pump inhibitors (PPIs) can restore chemosensitivity to GC cells, improving the response to antitumor drugs [88, 89]. One report demonstrated that omeprazole strengthens the activation of mTORC1 signaling by decreasing FTO expression in GC, restoring the chemosensitivity of tumor cells to chemotherapeutic drugs [90]. Further, omeprazole-mediated downregulation of FTO expression significantly increased overall m6A levels of GC cells, upregulating the expression of the apoptosis-related tumor suppressor gene, DDIT3, through m6A-dependent way, directly facilitating apoptosis of GC cells.

Therefore, FTO is of considerable clinical value for GC diagnosis and treatment. The regulation effect of target RNAs by FTO has been shown to both promote and inhibit.

### ALKBH5 in GC

Recently, the relationship between ALKBH5 and m6A methylation modification of lncRNAs in GC has garnered substantial consideration. Zhang et al. [91] demonstrated that ALKBH5 is highly expressed in GC and facilitates GC invasion and metastasis through the lncRNA NEAT1/EZH2 axis in an m6A-dependent manner. Further, the lncRNA NRON may be a regulatory subunit of ALKBH5 that can negatively regulate NANOG mRNA m6A modification to inhibit NANOG mRNA degradation, thus promoting GC cell proliferation [92].

In a more recent study [93] the demethylase, ALKBH5, was shown to inhibit GC invasion and migration by reducing PKMYT1 m6A levels, downregulating expression. ALKBH5 is poorly expressed in GC tissues and depends on its demethylase activity to play a tumor suppressor role in GC metastasis. Overexpression of ALKBH5 improves patient prognosis.

In conclusion, previous studies have shown ALKBH5 to be highly expressed in GC and to play a carcinogenic role. In contrast, ALKBH5 has also been shown to be downregulated in GC and to suppress GC progression. Therefore, the role of ALKBH5 in GC is controversial and further investigation is necessary to resolve this issue.

### YTHDC2 and YTHDF1/2 in GC

YTHDC2 and YTHDF1/2 are called m6A “Readers”, which identify and combine with the specific m6A sites on target mRNAs. Both YTHDC2 and YTHDF1 improve the translation efficiency of their targets through m6A. In contrast, YTHDF2 boosts the degradation of targets. Recently, the potential role of the three in GC has garnered significant interest.

Wei et al. [94] found YTHDC2 expression to be markedly increased in GC and to be related to a poor prognosis. YTHDC2-mediated m6A modification improves the translation efficiency of the oncogene YES-associated protein (YAP). Further, YAP/TEAD can directly and positively regulate YTHDC2 transcript expression, thus expanding the carcinogenic effect of YTHDC2/YAP and facilitating GC progression.

YTHDF1 is upregulated in GC and predicts unfavorable patient outcomes. Ubiquitin-specific protease 14 (USP14) is the m6A target of YTHDF1 in GC cells. YTHDF1-mediated m6A facilitates USP14 protein, thus, boosting GC cell proliferation, invasion, gastric tumorigenesis, and lung metastasis [95]. Furthermore, YTHDF1-dependent m6A enhances the expression of the Wnt receptor frizzled7 (FZD7), and facilitating GC proliferation and tumorigenesis via the Wnt/β-catenin pathway [46].

YTHDF2 is poorly expressed in GC and is known to inhibit GC progression. GC patients with low YTHDF2 expression levels have a reduced survival rate and a poor prognosis. YTHDF2 negatively regulates the mRNA stability of Forkhead box protein C2 (FOXC2) by recognizing its mRNA m6A modification sites, which restrains FOXC2 expression and suppresses cell proliferation, invasion, and migration of GC [96].

Existing literature shows that YTHDC2, YTHDF1, and YTHDF2 are explicitly involved in the initiation, progression, and prognosis of GC in an m6A-dependent manner. Among them, YTHDC2 and YTHDF1 are upregulated in GC and play oncogenic roles, while YTHDF2 is downregulated in GC and plays a tumor inhibitory effect.

### IGF2BP1/2/3 in GC

The mRNA expression of the m6A Reader proteins, IGF2BP1/2/3, is markedly elevated in GC. Moreover, high levels of IGF2BP1 mRNA are associated with poor overall survival of GC patients [97]. As indicated above, IGF2BP1/2/3 improve the stability and translation efficiency of their targets through an m6A-dependent manner, mediated by other m6A regulatory proteins.

IGF2BP1-METTL3-mediated m6A jointly increase SEC62 expression, thus facilitating GC proliferation [66]. In addition, IGF2BP1 directly targets c-MYC through an m6A-dependent pathway, enhancing mRNA stability and upregulating expression, which promotes GC cell migration, aerobic glycolysis, and tumor growth [98].

IGF2BP2 is highly expressed in GC and targets the mRNA of sirtuin 1 (SIRT1) in an m6A-dependent manner, affecting GC proliferation and migration [99]. Further, IGF2BP2 has been shown to elevate IGF1R expression by recognition of the specific m6A site on the mRNA of insulin-like growth factor 1 receptor (IGF1R), activating the RhoA-ROCK pathway, and promoting GC progression [100].

IGF2BP3 enhances PKMYT1 mRNA stability by identification and interaction with the m6A modification site, which upregulates expression and facilitating GC invasion and migration [93].

**Fig. 2:**
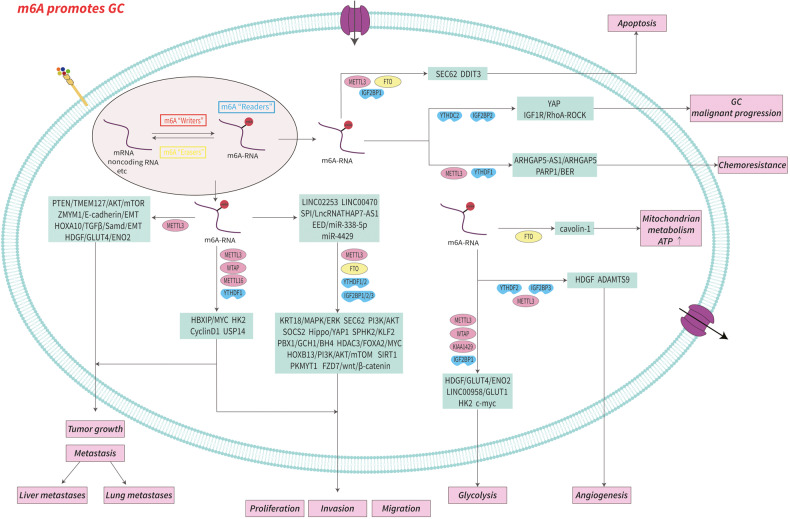
The mechanism and role of m6A in gastric cancer. m6A regulators promote the progression of gastric cancer by regulating the expression of related molecules in an m6A-dependent manner. See main text for more details.

**Fig. 3:**
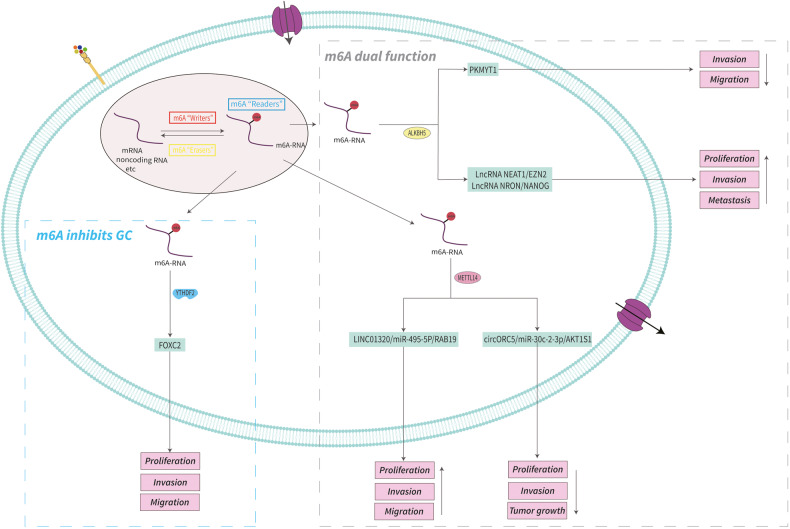
The mechanism and role of m6A in gastric cancer. YTHDF2 inhibits the progression of gastric cancer by regulating the expression of related molecules in an m6A-dependent manner. METTL14 and ALKBH5 promote or inhibit the progression of gastric cancer by regulating the expression of related molecules in an m6A-dependent manner.

In summary, m6A is fully involved in multiple biological processes of GC progression including proliferation, migration, invasion, tumor growth, drug resistance, angiogenesis, EMT, metastasis, cell mitochondrial metabolism, apoptosis, the Warburg effect, aerobic glycolysis (Figs. 2, 3), and is associated with patient prognosis. M6A may be a novel diagnostic modality and therapeutic target for GC, providing important insight into new strategies for GC treatment. Further, an understanding of m6A as an epigenetic marker for GC will provide a theoretical basis by which to improve the treatment of GC patients. In Table 2 are listed m6A regulatory proteins that function in GC in an m6A-dependent manner.

**Table 2:** The role of m6A regulators in gastric cancer^a,b^.

Regulator	Role in m6A modification	Function in gastric cancer	Abnormal change	Prognosis	Relevant mechanisms / targets	Reference
METTL3	Writer	Oncogene Promotes GC growth, proliferation, invasion, migration, chemoresistance, angiogenesis, metastasis, and EMT	Upregulated	Poor	METTL3/m6A/SOCS2 METTL3/m6A/YAP1 METTL3/m6A/PBX1/GCH1/BH4 LINC02253/METTL3/m6A/KRT18/MAPK/ERK miR-4429/METTL3-IGF2BP1/m6A/SEC62 SP1-METTL3-IGF2BP1/m6A/LncRNA THAP7-AS1/PI3K/AKT METTL3-YTHDF1/m6A/SPHK2/KLF2 ARHGAP5-AS1/METTL3/m6A/ARHGAP5 METTL3-YTHDF1/m6A/PARP1/BER METTL3-IGF2BP3/m6A/HDGF METTL3-YTHDF2/m6A/ADAMTS9 METTL3/m6A/HDGF/GLUT4/ENO2 METTL3/m6A/pri-miR17-92/PTEN/TMEM127/AKT/mTOR HBXIP/METTL3/m6A/MYC METTL3/m6A/ZMYM1-CtBP-LSD1-CoREST/E-cadherin/EMT HOXA10/METTL3/m6A/TGFβ/Smad/E-cadherin/EMT	[60] [61] [62] [65] [66] [67] [68] [69] [70] [71] [72] [71] [75] [76] [77] [78]
METTL14	Writer	Oncogene Promotes GC proliferation, invasion, and migration Suppressor Inhibits GC growth, proliferation, and invasion	unknown Downregulated	Poor Poor	METTL14/m6A/LINC01320/miR-495-5p/RAB19 METTL14/m6A/circORC5/miR-30c-2-3p/AKT1S1	[79] [80]
WTAP	Writer	Oncogene Promotes GC tumor growth, cell proliferation and Warburg effects	Upregulated	Poor	WTAP/m6A/HK2	[82]
KIAA1429 (VIRMA)	Writer	Oncogene Promotes GC cell aerobic glycolysis	Upregulated	Poor	KIAA1429/m6A/LINC00958/GLUT1	[83]
METTL16	Writer	Oncogene Promotes GC cell proliferation and tumor growth	Upregulated	Poor	METTL16/m6A/cyclinD1	[84]
FTO	Eraser	Oncogene Promotes GC proliferation, invasion, migration, tumor growth and metastasis, mitochondrial metabolism, and cell apoptosis	Upregulated	Poor	HDAC3/FOXA2/FTO/m6A/MYC FTO/m6A/HOXB13/PI3K/AKT/mTOR PPI/FTO/m6A/mTORC1/DDIT3 FTO/m6A/caveolin-1	[85] [86] [90] [87]
ALKBH5	Eraser	Oncogene Promotes GC proliferation, invasion, and metastasis	Upregulated	Poor	ALKBH5/m6A/lncRNA NEAT1/EZH2 ALKBH5-LncRNA NRON/m6A/NANOG	[91] [92]
		Suppressor Inhibits GC invasion and migration	Downregulated	Improved	ALKBH5/m6A/PKMYT1	[93]
YTHDC2	Reader	Oncogene Promotes GC progression	Upregulated	Poor	YTHDC2/m6A/YAP/YTHDC2 Positive feedback loop	[94]
YTHDF1	Reader	Oncogene Promote GC proliferation, invasion, tumorigenesis, and lung metastasis	Upregulated	Poor	YTHDF1/m6A/USP14 YTHDF1/m6A/FZD7/Wnt/β-catenin	[95] [46]
YTHDF2	Reader	Suppressor Inhibits GC proliferation, invasion, and migration	Downregulated	Improved	YTHDF2/m6A/FOXC2	[96]
IGF2BP1	Reader	Oncogene Promotes GC proliferation, migration, and aerobic glycolysis	Upregulated	Poor	IGF2BP1-METTL3/m6A/SEC62 IGF2BP1/m6A/c-MYC	[66] [98]
IGF2BP2	Reader	Oncogene Promotes GC proliferation and migration	Upregulated	Poor	IGF2BP2/m6A/SIRT1 IGF2BP2/m6A/IGF1R/RhoA-ROCK	[99] [100]
IGF2BP3	Reader	Oncogene Promotes GC invasion and migration	Upregulated	Poor	IGF2BP3/m6A/PKMYT1	[93]

^a^The role of m6A regulators which function in gastric cancer in an m6A-dependent manner is summarized.

^b^M6A regulators affect the progression of gastric cancer through the MYC pathway, Hippo pathway, MAPK/ERK pathway, PI3K/AKT/mTOR pathway, mTORC1 pathway, Wnt/β-catenin pathway, EMT pathway, and apoptosis pathway.

*GC* gastric cancer, *EMT* epithelial-mesenchymal transition.

## Clinical significance of m6A modification

New mechanisms and methods for diagnosis and prognosis are of particular importance to early diagnosis, treatment optimization, mortality reduction, and improved prognosis for GC patients.

Expression levels of m6A effectors are associated with the malignant clinicopathological characteristics of GC. In GC, the expression of WTAP, RBM15, and METTL3 are related to pathological stage. FTO and YTHDF3 expression are related to tumor stage. ALKBH5 expression is related to patient prognosis. YTHDC2 is related to patient survival [101]. One study found m6A methylation regulators to be useful as biomarkers for GC prognosis and recurrence. FTO is a significant oncogene in GC, is associated with EMT, and is a novel treated aim for EMT-alteration in GC [102]. FTO expression is correlated with the initiation and prognosis of GC, with high levels of FTO expression indicative of a poor prognosis for GC patients. FTO may be a critical target for the diagnosis and prognosis of GC [103–105]. GC expression levels of METTL3 [106], METTL14 [80], ALKBH1 [104], WTAP [105], IGF2BP1/2/3 [97], and METTL15 [107] relate to GC patient prognosis. A combined prediction model of FTO (oncogene) and RBM15 (protective gene) is an independent prognostic factor for GC. Clinicians can compute the corresponding risk value on the ground of the levels of RBM15 and FTO in GC, and thus, estimate patient outcomes with important clinical significance [108].

Surgery and combination chemotherapy are currently the main treatment modalities for patients with advanced GC. However, the high postoperative recurrence rate for patients with advanced GC [7] remains a serious clinical challenge. Therefore, postoperative follow-up is particularly important. One report indicated that postoperative m6A levels were markedly reduced in GC patients, indicating that m6A levels in peripheral blood RNA may be a latent and powerful biomarker for GC diagnosis and postoperative follow-up [109].

Since a critical role for m6A modification in GC tumorigenesis and progression has now been identified, attention has turned to cancer-targeted therapy directed at m6A [110]. Results have demonstrated the substrate, SAM, to be a cofactor necessary to methyl transfer by METTL3. Competitive inhibition of SAM effectively reduces the activity of the methyltransferase. Public METTL3 inhibitors are classified into two types: nucleosides and non-nucleoside. Both are substrate competitive inhibitors for the SAM pocket [111]. Nucleoside inhibitors include; adenosine 1 [112] (the first small molecule inhibitor of METTL3), one of the two moieties of SAM, and adenosine derivative compound 2 [111] (high inhibitory effect and ligand efficiency for METTL3). However, adenosine analogues (small molecule inhibitors) have some disadvantages such as poor cellular permeability and unsatisfactory efficacy as a single drug [113]. Therefore, other interventions are needed to enhance the efficacy of these small molecule inhibitors.

Non-nucleoside inhibitors include UZH1a [114] (a high-nanomolar inhibitor of METTL3 with selectivity and cell permeability), SAM adenosine part pocket filled with UZH1a, UZH2 [115] (an optimized UZH1a analogue) with significantly improved solubility and metabolic stability and selective inhibition of METTL16 and METTL1 compared to UZH1a, and STM2457 [116] (a highly efficient and selective METTL3 catalytic inhibitor) that binds to SAM binding sites and exerts its anti-AML effect without markedly affecting normal hematopoiesis. Further, multiple inhibitors targeting FTO such as meclofenamic acid (MA) [117], R-2-Hydroxyglutarate (R-2HG) [118], FB23, and FB23-2 [119], exert anti-cancer effects by selectively inhibiting the activity of FTO. In addition, hematopoietic transcription factor SPI1 [120] can be targeted to inhibit the expression of METTL14 in malignant hematopoietic cells and may assist in the treatment of AML. Carbononic anhydrase IV (CA4) [121] inhibits the tumorigenicity of colorectal cancer (CRC) by targeting inhibition of WTAP.

Nevertheless, the studies of such subject are restricted. Especially in GC, there are almost no relevant reports of m6A-targeted inhibition. Such targeted inhibition may become a significant research direction for GC therapy in the future.

## Conclusions and future perspectives

The purpose of this review was to summarize the detection methods and the chemical basis for m6A epigenetic modifications, the physiological functions and targeted inhibitors of m6A-related effectors. In addition, we have focused on the potential roles and clinical applications of various regulators associated with m6A modifications in GC. Epigenetics is a current focus of cancer mechanism research, with m6A methylation important to an understanding of RNA editing and modification in GC. Understanding m6A mechanisms may identify the biological basis for GC progression and may offer fresh targets for the diagnosis and therapy of GC. The process of m6A methylation is complex and m6A effectors affect RNA expression by playing their respective functional roles. A single m6A regulatory factor can act alone or interact with other related regulatory factors to jointly affect the occurrence and progress of GC. Dysregulation of m6A methylation is associated with abnormalities in the MYC pathway, Hippo pathway, MAPK/ERK pathway, PI3K/AKT/mTOR pathway, mTORC1 pathway, Wnt/β-catenin pathway, EMT pathway, and apoptosis pathway in GC. Additionally, m6A-dependent methylation regulates the processing, splicing, and translation of noncoding RNAs (including microRNAs, circRNAs, and pri-miRNAs et al.), thus affecting the expression and function of non-coding RNAs in GC.

The m6A regulators (RBM15, HAKAI/CBLL1, ZC3H13, METTL5, METTL7A, SMAD2/3, CEBPZ, ALKBH1, HNRNPA2/B1, HNRNPC/G, YTHDF3, and EIF3) are associated with GC, but have not been reported for their explicit involvement in GC progression in an m6A-dependent manner.

Predictive studies of prognostic risk and tumor recurrence for GC patients have mainly focused on the demethylase, FTO. Few studies have evaluated other related factors. Moreover, current targeted inhibitors of METTL3, FTO, and METTL14 are primarily used in the clinical treatment of other cancers (such as leukemia). As far as we know, there are no relevant reports of m6A targeted inhibitors used for the clinical treatment of GC.

Although we have some understanding of m6A in GC, our knowledge is limited. Therefore, further investigation of m6A is needed to explore novel approaches to GC diagnosis and prognosis, and to develop new targets for GC treatment. In this manner, early diagnosis of GC will be improved and the prognosis for GC patients enriched.

In conclusion, we summarized existing issues and future research directions for a preferably comprehending of the function of m6A in GC. The roles of METTL14 and ALKBH5 in GC remain controversial and require resolution. The development of targeted inhibitors of FTO, METTL3, and other Reader proteins needs to be the focus of GC targeted treatment research in the future. Furthermore, the biological function of m6A regulators in GC must be identified, as do their upstream and downstream target genes in order to systematically understand the underlying mechanistic role for m6A in GC. Addressing these issues and future research directions will improve outcomes for GC patients.

## Data Availability

The studies included were retrieved from the PubMed database.
